# Ultrasound biomicroscopy-based quantitative assessment of anterior segment biometric changes in contralateral eyes of unilateral neovascular glaucoma: a retrospective case-control study

**DOI:** 10.3389/fmed.2026.1722824

**Published:** 2026-02-17

**Authors:** Fang Lin, Yuwei You, Yuxing Su

**Affiliations:** Xinjiang 474 Hospital, Ürümqi, Xinjiang, China

**Keywords:** age stratification, anterior segment parameters, fellow eye, neovascular glaucoma, risk assessment, ultrasound biomicroscopy

## Abstract

**Background:**

Neovascular glaucoma (NVG) is a blinding ocular disease secondary to severe retinal ischemic disorders, characterized by rapid progression and severe visual impairment. While most studies focus on diagnosis and intervention of affected eyes in NVG, systematic evaluation of structural status and potential risks in fellow eyes remains insufficient.

**Objective:**

This study aims to quantitatively analyze anterior segment structures in fellow eyes of unilateral NVG patients using ultrasound biomicroscopy (UBM), identify differences from normal eyes, and investigate age-related variations in anterior segment parameters along with their diagnostic efficacy.

**Methods:**

This retrospective case-control study included fellow eyes of unilateral NVG patients (observation group) and age- and gender-matched normal eyes (control group). UBM-measured parameters included anterior chamber depth (ACD), angle opening distance (AOD500/750), trabecular-iris angle (TIA500/750), iris thickness (IT500/750/2000), iris convexity (IC), trabecular-ciliary process distance (TCPD), and iris-cornea posterior surface distance (ICPD). Age-stratified analysis, parameter correlation, and ROC curve evaluation were performed.

**Results:**

Compared with controls, the observation group showed significantly shallower anterior chambers (reduced ACD), narrower angles (decreased AOD/TIA), and posterior iris bowing (elevated IC) (all *p* < 0.05). These differences were more pronounced in elderly subgroups, with IC, ACD, and AOD750 demonstrating strong discriminative power in ROC analysis (AUC > 0.7). Correlation analysis revealed AOD500 was positively associated with ACD and TIA but negatively with IC and IT750, suggesting interconnected angle anatomy modulated by age.

**Conclusion:**

Fellow eyes of unilateral NVG patients exhibit multiple abnormal anterior segment alterations despite being clinically unaffected, particularly in the elderly. UBM provides reliable quantitative indicators for early high-risk identification, with IC, ACD, and AOD750 serving as potential warning parameters, laying a theoretical foundation for risk screening models and preventive strategies.

## 1 Introduction

Neovascular glaucoma (NVG) is a severe secondary glaucoma triggered by ocular or systemic ischemic diseases, characterized by rapid progression, sustained elevated intraocular pressure (IOP), and irreversible visual impairment, ultimately leading to blindness (1–3). Primary causative factors include diabetic retinopathy (DR), retinal vein occlusion (RVO), and ocular ischemic syndrome, all of which stimulate vascular endothelial growth factor (VEGF) release, inducing iris and angle neovascularization, thereby disrupting angle structure and impeding aqueous outflow (4–7). Although NVG predominantly manifests unilaterally, accumulating clinical evidence suggests that fellow eyes may exhibit subtle anterior segment abnormalities prior to overt clinical onset. These changes may stem from ocular microcirculatory disturbances, systemic metabolic factors, or shared binocular anatomical features (8–10). Conventional diagnostic tools (gonioscopy, slit-lamp examination) often fail to detect early microstructural alterations. In contrast, UBM, with its high resolution and penetration depth, provides detailed visualization of corneal, iris, ciliary body, lens, and angle structures, offering superior capability for identifying early anterior segment abnormalities (11–13). Previous UBM research has primarily focused on conditions like primary angle-closure glaucoma (PACG), where its value in early diagnosis, subtyping, and preoperative assessment is well-established (14, 15). However, systematic quantitative analyses of anterior segment changes in fellow eyes of NVG patients remain scarce. This study employs UBM to quantitatively analyze anterior segment parameters in fellow eyes of unilateral NVG patients, comparing them with age-matched normal controls. By elucidating these structural alterations, we aim to explore their clinical implications and establish an evidence-based foundation for early risk identification, screening protocols, and intervention strategies—ultimately mitigating NVG-related blindness and improving patient outcomes.

## 2 Materials and methods

### 2.1 Study design and participants

This retrospective case-control study compared anterior segment structures between fellow eyes of unilateral NVG patients and normal controls. Participants were recruited from the 474th Hospital of Xinjiang between January 2022 and June 2023, with data sourced from electronic medical records and imaging archives.

Study group (fellow eyes of NVG): included 52 fellow eyes from unilateral NVG patients (52 eyes). NVG diagnosis in the affected eyes adhered to the 2019 Chinese Expert Consensus on Diagnosis and Management of NVG, with primary etiologies being DR and RVO. Fellow eyes showed no clinical abnormalities, with preserved visual acuity and normal IOP.

Control group (normal eyes): comprised 35 eyes from 22 healthy volunteers without glaucoma family history, ocular diseases, or abnormal anterior segment structures (IOP 10–21 mmHg). Controls were age- and gender-matched to ensure baseline comparability.

Inclusion criteria: (1) age 50–70 years; (2) For study group: unilateral NVG diagnosis with clinically normal fellow eyes; (3) For controls: no glaucoma-related ocular or systemic diseases; (4) Best-corrected visual acuity ≥0.2 and IOP 10–21 mmHg; (5) Cooperative for UBM examination with complete, clear imaging data. Exclusion criteria: (1) Comorbidities affecting anterior segment anatomy (e.g., glaucoma, cataract, iritis, ocular trauma); (2) History of ocular surgery; (3) Poor-quality UBM images precluding parameter measurement; (4) Severe systemic metabolic/psychiatric disorders impairing examination compliance. The study complied with the Declaration of Helsinki and was approved by the Institutional Ethics Committee.

### 2.2 Examination methods

All participants underwent UBM examinations performed by the same experienced technician using the same device to ensure data consistency and reproducibility. Prior to the examination, all subjects received routine ophthalmic assessments, including visual acuity measurement, IOP monitoring (using non-contact tonometry), slit-lamp microscopy, and fundus examination to exclude significant ocular pathologies. To account for the potential impact of myopia and hyperopia on anterior segment anatomy, we also measured refractive errors and axial length in all participants. Refractive errors were assessed using standard refraction methods, and axial length was measured using an optical biometer (IOLMaster, Carl Zeiss Meditec, Germany).

#### 2.2.1 Examination apparatus and imaging protocol

Apparatus model: the UBM instrument (Registration No.: National Medical Device Approval 20163161075; Model: MD-320W) manufactured by Tianjin MEDA Medical Technology Co., Ltd. was employed.

Operating method: imaging was performed using the “liquid-bath technique” with the patient in a supine position while maintaining eye opening.

Imaging procedure: (1) Instillation of topical anesthetic (0.4% oxybuprocaine); (2) Placement of an eyelid speculum between the eyelids; (3) Positioning of a dedicated eye cup with sterile saline solution filling the chamber; (4) Sequential acquisition of cross-sectional images from four quadrants (nasal, temporal, superior, and inferior); (5) Continuous capture of three images per quadrant, with the middle image selected for parametric measurement and analysis. During examination, meticulous care was taken to avoid globe compression or induction of IOP fluctuations. All imaging data were independently evaluated by two physicians, and images with higher inter-observer agreement were used for subsequent analysis.

#### 2.2.2 Anterior segment measurement parameters

In the UBM images, the anatomical structures of the anterior segment were clearly distinguishable. All parameters were measured by the same experienced imaging specialist using the built-in measuring tools of the device to ensure data consistency. Each parameter was measured three times consecutively in each of the four quadrants (nasal, temporal, superior, and inferior), and the average of these three measurements was used for statistical analysis (see Table 1).

**Table 1:** Anterior segment structural parameters.

Abbreviation	Parameter name	Definition
ACD	Anterior chamber depth	The vertical distance from the central corneal endothelium to the anterior surface of the lens. Reflects the overall depth of the anterior chamber and is a key indicator of angle closure tendency.
AOD500/AOD750	Angle opening distance at 500 μm/750 μm	The distance from the angle recess (Schwalbe’s line) perpendicularly to the anterior iris surface at points 500 μm and 750 μm posteriorly. Reflects the width of the anterior chamber angle.
TIA500/TIA750	Trabecular-iris angle at 500 μm/750 μm	The angle formed at the angle recess between the corneal endothelium and the iris surface at points 500 μm and 750 μm posteriorly. Together with AOD, it reflects the degree of angle opening.
IT500/IT750/IT2000	Iris thickness at 500 μm/750 μm/2000 μm	The thickness of the iris measured at points 500 μm, 750 μm, and 2000 μm from the angle recess. Evaluates iris thickening and forward displacement trends.
IC	Iris convexity	The vertical distance from the iris root to the most anteriorly convex point on the iris surface. This parameter reflects the curvature of the iris and is closely related to iris bombe and angle closure.
TCPD	Trabecular-ciliary process distance	The shortest linear distance between the posterior trabecular meshwork and the anterior edge of the ciliary process. Reflects the relative position and anterior displacement trend of the ciliary body.
ICPD	Iris-ciliary process distance	The shortest distance between the posterior iris surface and the anterior edge of the ciliary process. Evaluates the anatomical proximity of the iris–ciliary process complex.

Measurement protocol: all quantitative parameters were measured using the linear measurement tools integrated into the UBM analysis software. Each parameter was measured three times consecutively, with the mean value adopted as the final statistical datum. To minimize bias, researchers performing image analysis were blinded to group allocation during measurements. Images from any quadrant failing to meet predefined quality criteria (e.g., inadequate resolution or artifact interference) were excluded from the final calculation of quadrant-based averages.

#### 2.2.3 Image quality control

To ensure the accuracy and reproducibility of anterior segment parameter measurements, this study implemented strict quality control measures throughout the UBM image acquisition and evaluation process, as detailed below:

(1) Examination procedure consistency: all UBM examinations were performed by the same qualified and experienced technician. Prior to imaging, topical anesthesia was administered using 0.4% oxybuprocaine eye drops to minimize eyelid pressure artifacts. A standardized fluid-bath method was applied for all participants, with images acquired sequentially from the superior, inferior, nasal, and temporal quadrants to ensure procedural uniformity.
(2) Image acquisition criteria: three consecutive images were captured per quadrant. The image analyst selected the one with optimal quality for parameter measurement. Images were required to clearly depict key anatomical structures including the cornea, anterior chamber, angle recess, iris, and ciliary processes. Images with blurred boundaries, distortions, or obstructions were deemed invalid.
(3) Image evaluation and measurement consistency: all measurements were performed independently by two imaging physicians experienced in ophthalmic image analysis. If the discrepancy between their measurements for any parameter was less than 10%, the average value was adopted. If the discrepancy exceeded 10%, a third senior physician re-evaluated and re-measured the image to determine the final value. All evaluators were blinded to group assignments to avoid observer bias.
(4) Strict exclusion criteria: if images from any quadrant failed to meet quality standards, data from that quadrant were excluded from analysis. Eyes with fewer than two valid quadrants were entirely excluded from the study. All data underwent a final item-by-item review by a supervisor prior to database entry to ensure authenticity, completeness, and reliability.

### 2.3 Statistical analysis

All data were processed using SPSS Statistics 26.0. Continuous variables were first tested for normality via the Kolmogorov-Smirnov test. Normally distributed data are expressed as mean ± standard deviation (*x̄* ± *s*), and intergroup comparisons were performed using independent samples *t*-tests. Non-normally distributed data are presented as median and interquartile range, and compared using the Mann–Whitney *U* test. Categorical variables were analyzed using Chi-square tests or Fisher’s exact test, as appropriate. To control for the potential confounding effect of age, both experimental and control groups were further stratified into two subgroups: 50–60 years and 60–70 years. A two-way ANOVA was employed to evaluate the interaction between group membership and age. Correlations among major anterior segment parameters were assessed using Pearson or Spearman correlation coefficients, depending on the distribution of variables. Key parameters showing statistically significant differences were subjected to receiver operating characteristic (ROC) curve analysis to evaluate their discriminative power in identifying potential structural abnormalities in contralateral eyes of NVG patients. All tests were two-sided, and a *p*-value < 0.05 was considered statistically significant.

## 3 Results

### 3.1 Comparison of baseline characteristics

This study included 52 unaffected fellow eyes of unilateral NVG patients in the observation group and 35 age- and gender-matched healthy individuals in the control group. The two groups showed good comparability in demographic characteristics. Overall, the mean age was 61.37 ± 7.287 years in the observation group and 59.03 ± 6.623 years in the control group, with no statistically significant difference (*p* = 0.113). The male-to-female ratio was 29/23 in the observation group and 17/18 in the control group (*p* = 0.825), also showing no significant difference. However, statistically significant differences were observed in visual function and optic nerve structural parameters. The observation group exhibited significantly worse LogMAR visual acuity [0.7 (0.4) vs. 0.4 (0.2), *p* < 0.05], suggesting potential visual impairment. Additionally, the cup-to-disc ratio was significantly larger in the observation group [0.30 (0.2) vs. 0.2 (0.1), *p* < 0.05], indicating structural optic nerve changes possibly associated with NVG. IOP was higher in the observation group (16.69 ± 3.215 mmHg vs. 15.46 ± 3.100 mmHg), though the difference did not reach statistical significance (*p* = 0.078). Stratified analysis by age revealed that among younger participants (≤60 years), there were no significant differences in age (55.20 ± 3.136 vs. 54.92 ± 5.244, *p* = 0.774) or gender distribution (15/9 vs. 10/7, *p* = 0.113) between the two groups. However, the observation group still demonstrated significantly worse LogMAR visual acuity [0.6 (0.3) vs. 0.3 (0.2)] and larger cup-to-disc ratios [0.30 (0.1) vs. 0.2 (0.1)] (*p* < 0.05). IOP showed a trend toward elevation (16.64 ± 3.144 mmHg vs. 15.34 ± 2.963 mmHg), approaching statistical significance (*p* = 0.053). In the older subgroup (>60 years), the observation group had slightly higher mean age (67.07 ± 4.969 vs. 64.41 ± 3.543), though the difference was not statistically significant (*p* = 0.081). Gender distribution also did not differ (14/14 vs. 7/11, *p* = 0.927). Visual acuity and cup-to-disc ratio remained significantly worse in the observation group [visual acuity 0.8 (0.5) vs. 0.5 (0.2); cup-to-disc ratio 0.30 (0.3) vs. 0.2 (0.1); all *p* < 0.05]. IOP demonstrated no significant difference (16.74 ± 3.457 mmHg vs. 15.47 ± 3.205 mmHg, *p* = 0.661). In both the younger and older age groups, the differences in refractive error were small and not statistically significant (*p* > 0.05), indicating that the impact of refractive error on anterior segment anatomy is similar across different age groups. In this study, there were no significant differences in refractive error and axial length between the observation and control groups, either in the younger (≤60 years) or older (>60 years) groups (*p* > 0.05). This suggests that the impact of refractive error and axial length on anterior segment anatomy is relatively small across different age groups (see Table 2).

**Table 2:** Comparison of baseline characteristics of subjects.

Item	Overall			Young group (≤60 years)			Old group (>60 years)		
	Observation group (*n* = 52)	Control group (*n* = 35)	*p*	Observation group (*n* = 24)	Control group (*n* = 17)	*p*	Observation group (*n* = 28)	Control group (*n* = 18)	*p*
Age (years)	61.37 ± 7.287	59.03 ± 6.623	0.113	55.20 ± 3.136	54.92 ± 5.244	0.774	67.07 ± 4.969	64.41 ± 3.543	0.081
Gender (male/female)	29/23	17/18	0.510	15/9	10/7	0.812	14/14	7/11	0.460
Visual acuity (LogMAR)	0.7(0.4)	0.4 (0.2)	<0.05	0.6 (0.3)	0.3 (0.2)	<0.05	0.8 (0.5)	0.5 (0.2)	<0.05
IOP (mmHg)	16.69 ± 3.215	15.46 ± 3.100	0.078	16.64 ± 3.144	15.34 ± 2.963	0.053	16.74 ± 3.457	15.47 ± 3.205	0.661
C/D ratio	0.30 (0.2)	0.2 (0.1)	<0.05	0.30 (0.1)	0.2 (0.1)	<0.05	0.30 (0.3)	0.2 (0.1)	<0.05
Refractive error (refractive power)	−0.75 ± 1.12	−0.68 ± 1.03	0.639	−0.77 ± 1.15	−0.65 ± 1.10	0.572	−0.73 ± 1.10	−0.72 ± 1.05	0.885
Axial length (mm)	23.56 ± 1.28	23.64 ± 1.31	0.71	23.47 ± 1.32	23.53 ± 1.27	0.839	23.63 ± 1.24	23.76 ± 1.33	0.783

### 3.2 Overall comparison of anterior segment parameters

Quantitative measurement and comparison of anterior segment structural parameters using UBM revealed statistically significant differences between the observation and control groups in multiple key angle and iris parameters, suggesting that although clinically presenting with normal IOP, the fellow eyes of unilateral NVG patients already exhibit concealed anatomical remodeling changes. Regarding ACD, the ACD in the observation group (2.323 ± 0.606 mm) was significantly shallower than in the control group (2.755 ± 0.290 mm), with this difference being statistically significant (*t* = 4.45, *p* < 0.05), indicating an overall reduction in aqueous chamber volume. For anterior chamber angle openness indicators, the median AOD500 and AOD750 in the observation group were 0.247 mm and 0.330 mm respectively, significantly lower than those in the control group (0.315 and 0.458 mm), with *Z* values of −2.931 and −2.987 respectively, showing statistically significant differences (*p* < 0.05). Similarly, the observation group also demonstrated significantly narrower anterior chamber angles in TIA500 and TIA750 parameters (26.221° and 26.500° vs. 33.669° and 35.631°, *Z* = −3.437 and −3.566, *p* < 0.05), reflecting restricted openness of the corneal limbal aqueous drainage pathway in fellow eyes. Regarding IT indicators, the observation group showed significantly greater IT750 (0.400 ± 0.084 mm) than the control group (0.346 ± 0.040 mm, *p* < 0.05), while IT500 and IT2000 measurements, although numerically higher, did not reach statistical significance (*p* = 0.058 and *p* = 0.186). These results suggest that localized thickening may exist in the peripheral iris region, possibly participating in or exacerbating angle closure tendency. For the distance parameter from the angle to the ciliary process, the IC in the observation group was significantly greater than in the control group (0.240 ± 0.112 mm vs. 0.128 ± 0.027 mm, *p* < 0.05), suggesting anatomical remodeling of the spatial relationship between the iris root and ciliary body tissue. Neither TCPD nor ICPD showed significant differences between the two groups (*p* = 0.119 and *p* = 0.334), indicating no obvious structural changes in the ciliary process region (see Table 3).

**Table 3:** Overall comparison of anterior segment parameters between the observation group and the control group.

Parameter name	Observation group	Control group	*t*	*Z*	*p*
ACD (mm)	2.323 ± 0.606	2.755 ± 0.290	4.45	—	<0.05
AOD500 (mm)	0.247 (0.184)	0.315 (0.056)	—	−2.931	<0.05
AOD750 (mm)	0.330 (0.031)	0.458 ± 0.094	—	−2.987	<0.05
TIA500 (°)	26.221 (17.8)	33.669 (11.8)	—	−3.437	<0.05
TIA750 (°)	26.500 (17.3)	35.631 ± 7.147	—	−3.566	<0.05
IT500 (mm)	0.372 ± 0.077	0.347 ± 0.047	1.92	—	0.058
IT750 (mm)	0.400 ± 0.084	0.346 ± 0.040	4	—	<0.05
IT2000 (mm)	0.481 ± 0.077	0.461 ± 0.060	1.334	—	0.186
IC (mm)	0.240 ± 0.112	0.128 ± 0.027	6.91	—	<0.05
TCPD (mm)	0.320 (0.32)	0.412 ± 0.069	—	−1.559	0.119
ICPD (mm)	0.245 (0.22)	0.293 ± 0.075	—	−0.966	0.334

### 3.3 Age-stratified subgroup analysis

#### 3.3.1 Comparison between younger observation group and younger control group

The younger observation group and younger control group demonstrated statistically significant differences in certain anterior segment parameters. The observation group showed significantly lower values in TIA parameters TIA500 and TIA750 compared to controls [30.900° (IQR: 16.7) vs. 38.565 ± 7.089°, *p* < 0.05; 32.426 ± 13.485° vs. 41.335 ± 6.936°, *p* < 0.05], indicating restricted anterior chamber angle opening. Additionally, IT parameter IT750 was significantly greater in the observation group (0.388 ± 0.082 mm vs. 0.346 ± 0.040 mm, *p* < 0.05). Although parameters including ACD, AOD500, and AOD750 showed decreasing trends in the observation group, these differences did not reach statistical significance (*p* > 0.05). A notably greater iridociliary distance (IC) was observed in the study group (0.226 ± 0.106 mm vs. 0.128 ± 0.027 mm, *p* < 0.05), suggesting possible posterior displacement of the iris root or structural remodeling (see Table 4).

**Table 4:** Comparison of anterior segment parameters between the young observation group and the young control group.

Parameter name	Young observation group	Young control group	*t*	*Z*	*p*
ACD (mm)	2.492 ± 0.715	2.801 ± 0.341	−1.90	—	0.065
AOD500 (mm)	0.330 ± 0.134	0.335 ± 0.054	−0.16	—	0.872
AOD750 (mm)	0.425 ± 0.206	0.498 ± 0.096	−1.56	—	0.127
TIA500 (°)	30.900 (16.7)	38.565 ± 7.089	—	−2.319	<0.05
TIA750 (°)	32.426 ± 13.485	41.335 ± 6.936	−2.384	—	<0.05
IT500 (mm)	0.367 ± 0.074	0.347 ± 0.057	1.17	—	0.253
IT750 (mm)	0.388 ± 0.082	0.346 ± 0.040	2.02	—	<0.05
IT2000 (mm)	0.490 ± 0.079	0.461 ± 0.060	1.34	—	0.184
IC (mm)	0.226 ± 0.106	0.128 ± 0.027	4.28	—	<0.05
TCPD (mm)	0.320 (0.32)	0.412 ± 0.068	—	−0.748	0.455
ICPD (mm)	0.260 (0.34)	0.306 ± 0.088	—	−0.432	0.672

#### 3.3.2 Comparison between older observation group and older control group

Results in the older age group demonstrated that the observation group had significantly shallower ACD compared to controls (2.165 ± 0.441 mm vs. 2.694 ± 0.290 mm, *p* < 0.05). All anterior chamber angle parameters, including AOD500, AOD750, TIA500, and TIA750, were significantly smaller in the observation group (*p* < 0.05), indicating more severely restricted anterior chamber angle openness in the older observation eyes. IT750 was significantly thicker in the observation group (0.411 ± 0.085 mm vs. 0.341 ± 0.049 mm, *p* < 0.05), suggesting overall iris thickening. IC was also significantly greater in the observation group (0.251 ± 0.114 mm vs. 0.127 ± 0.028 mm, *p* < 0.05). However, no significant differences were observed in parameters including IT500, IT2000, TCPD, and ICPD (*p* > 0.05), indicating no obvious changes in posterior segment supporting structures (see Table 5).

**Table 5:** Comparison of anterior segment parameters between the old observation group and the old control group.

Parameter name	Old observation group	Old control group	*t*	*Z*	*p*
ACD (mm)	2.165 ± 0.441	2.694 ± 0.290	−5.32	—	<0.05
AOD500 (mm)	0.201 (0.136)	0.288 ± 0.048	—	−3.374	<0.05
AOD750 (mm)	0.270 (0.227)	0.404 ± 0.059	—	−2.823	<0.05
TIA500 (°)	21.174 ± 10.690	27.140 ± 2.297	−2.79	—	<0.05
TIA750 (°)	22.763 ± 10.950	30.254 ± 6.936	−3.12	—	<0.05
IT500 (mm)	0.374 ± 0.079	0.348 ± 0.037	1.43	—	0.165
IT750 (mm)	0.411 ± 0.085	0.341 ± 0.049	3.09	—	<0.05
IT2000 (mm)	0.471 ± 0.077	0.460 ± 0.061	0.54	—	0.59
IC (mm)	0.251 ± 0.114	0.127 ± 0.028	5.21	—	<0.05
TCPD (mm)	0.320 (0.32)	0.413 ± 0.070	—	−1.596	0.111
ICPD (mm)	0.255 ± 0.30	0.280 ± 0.053	—	−0.432	0.672

#### 3.3.3 Comparison between younger and older observation groups

Intra-group comparisons across different age strata revealed that the younger group had significantly greater AOD500 and AOD750 values than the older group [0.330 ± 0.134 mm vs. 0.201 mm (IQR: 0.136), *p* < 0.05; 0.425 ± 0.206 mm vs. 0.270 mm (IQR: 0.227), *p* < 0.05]. Similarly, TIA500 and TIA750 were significantly larger in the younger group (*p* < 0.05), indicating more pronounced angle compression in older subjects. The ACD was significantly deeper in the younger group (2.492 ± 0.715 mm vs. 2.165 ± 0.441 mm, *p* < 0.05), reflecting progressive anterior chamber shallowing with advancing age. Notably, parameters including IT500, IT750, IT2000, IC, and ICPD showed no significant differences between age groups (*p* > 0.05). Only TCPD was greater in the younger group (0.438 ± 0.211 mm vs. 0.320 mm (IQR: 0.32), *p* < 0.05), possibly suggesting more robust ciliary body support or less degenerative changes in younger individuals (see Table 6).

**Table 6:** Comparison of anterior segment parameters between the young observation group and the old observation group.

Parameter name	Young observation group	Old observation group	*t*	*Z*	*p*
ACD (mm)	2.492 ± 0.715	2.165 ± 0.441	2.01	—	<0.05
AOD500 (mm)	0.330 ± 0.134	0.201 (0.136)	—	−3.591	<0.05
AOD750 (mm)	0.425 ± 0.206	0.270 (0.227)	—	−2.308	<0.05
TIA500 (°)	30.900 (16.7)	21.174 ± 10.690	—	−2.784	<0.05
TIA750 (°)	33.316 ± 15.067	22.763 ± 10.950	2.963	—	<0.05
IT500 (mm)	0.367 ± 0.074	0.374 ± 0.079	−0.48	—	0.636
IT750 (mm)	0.388 ± 0.082	0.411 ± 0.085	−1.07	—	0.293
IT2000 (mm)	0.490 ± 0.079	0.471 ± 0.077	0.88	—	0.382
IC (mm)	0.226 ± 0.106	0.251 ± 0.114	−0.96	—	0.342
TCPD (mm)	0.438 ± 0.211	0.320 (0.32)	—	−2.428	<0.05
ICPD (mm)	0.260 (0.34)	0.255 ± 0.30	—	−0.752	0.452

### 3.4 Correlation analysis of anterior segment parameters

**Figure 1:**
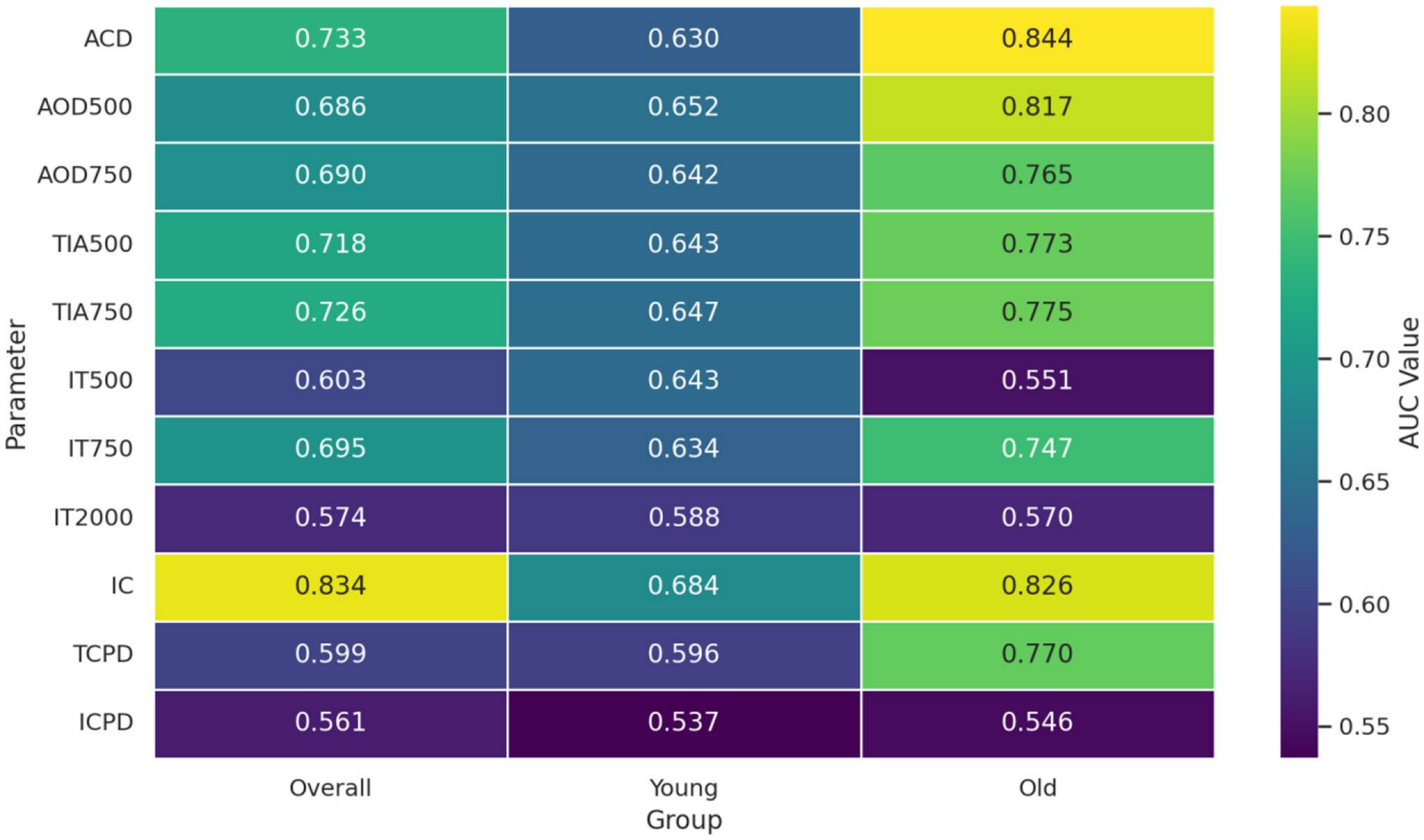
Heat map of the diagnostic performance (AUC) of anterior segment parameters for identifying fellow eyes with NVG in different age subgroups.

To investigate the central role of anterior chamber angle openness in the structural alterations of fellow eyes in NVG, this study selected angle opening distance (AOD500) as the core variable and analyzed its correlations with other anterior segment parameters in the overall observation group, younger subgroup (≤60 years), and older subgroup (>60 years). In the overall observation group, AOD500 exhibited moderate positive correlations with ACD (*r* = 0.593, *p* < 0.05), and strong positive correlations with AOD750, TIA500, and TIA750 (*r* = 0.799, 0.867, and 0.838, respectively; all *p* < 0.05), indicating good consistency of angle openness in anterior chamber assessments. Additionally, AOD500 showed significant negative correlations with IT750 (*r* = −0.308, *p* < 0.05) and IC (*r* = −0.465, *p* < 0.05), suggesting that iris thickening and posterior displacement of the iris root may contribute to anterior chamber angle narrowing. In the younger subgroup, AOD500 maintained moderate-to-strong positive correlations with ACD (*r* = 0.492, *p* < 0.05), AOD750 (*r* = 0.774, *p* < 0.05), TIA500 (*r* = 0.808, *p* < 0.05), and TIA750 (*r* = 0.798, *p* < 0.05). Although negative correlations were observed with IT750 (*r* = −0.267, *p* = 0.196) and IC (*r* = −0.325, *p* = 0.113), these did not reach statistical significance, implying stronger anatomical compensatory ability in younger individuals with less pronounced structural variations. In the older subgroup, AOD500 showed an even stronger correlation with ACD (*r* = 0.653, *p* < 0.05), and its positive correlations with AOD750, TIA500, and TIA750 were significantly enhanced (*r* = 0.834, 0.892, and 0.884, respectively; all *p* < 0.05), suggesting greater consistency among anterior angle parameters in elderly individuals. A significant negative correlation was observed with IT750 (*r* = −0.378, *p* < 0.05), indicating that IT exerts more pronounced compression on angle openness in advanced age. Additionally, a borderline significant negative correlation was noted for IC (*r* = −0.358, *p* = 0.067), implying increased anterior displacement of the iris root in older subjects. Other parameters such as TCPD and ICPD showed weak, non-significant correlations with AOD500, suggesting that ciliary body support structures may not significantly influence angle openness at this stage. Further validation with longitudinal dynamic data may be required (see Table 7 and Figure 1).

**Table 7:** Correlation analysis between AOD500 and other anterior segment parameters in different age subgroups of the observation group.

Parameter	Observation group		Young observation group		Old observation group	
	*r*	*p*	*r*	*p*	*r*	*p*
ACD	0.593	<0.05	0.492	<0.05	0.653	<0.05
AOD500	1	—	1	—	1	—
AOD750	0.799	<0.05	0.774	<0.05	0.834	<0.05
TIA500	0.867	<0.05	0.808	<0.05	0.892	<0.05
TIA750	0.838	<0.05	0.798	<0.05	0.884	<0.05
IT500	−0.204	0.148	−0.164	0.295	−0.152	0.449
IT750	−0.308	<0.05	−0.267	0.196	−0.378	<0.05
IT2000	−0.004	0.979	−0.176	0.4	0.007	0.973
IC	−0.465	<0.05	−0.325	0.113	−0.358	0.067
TCPD	0.221	0.115	0.239	0.249	0.048	0.813
ICPD	0.04	0.779	−0.192	0.358	0.070	0.728

### 3.5 Diagnostic performance evaluation by ROC curve analysis

**Figure 2:**
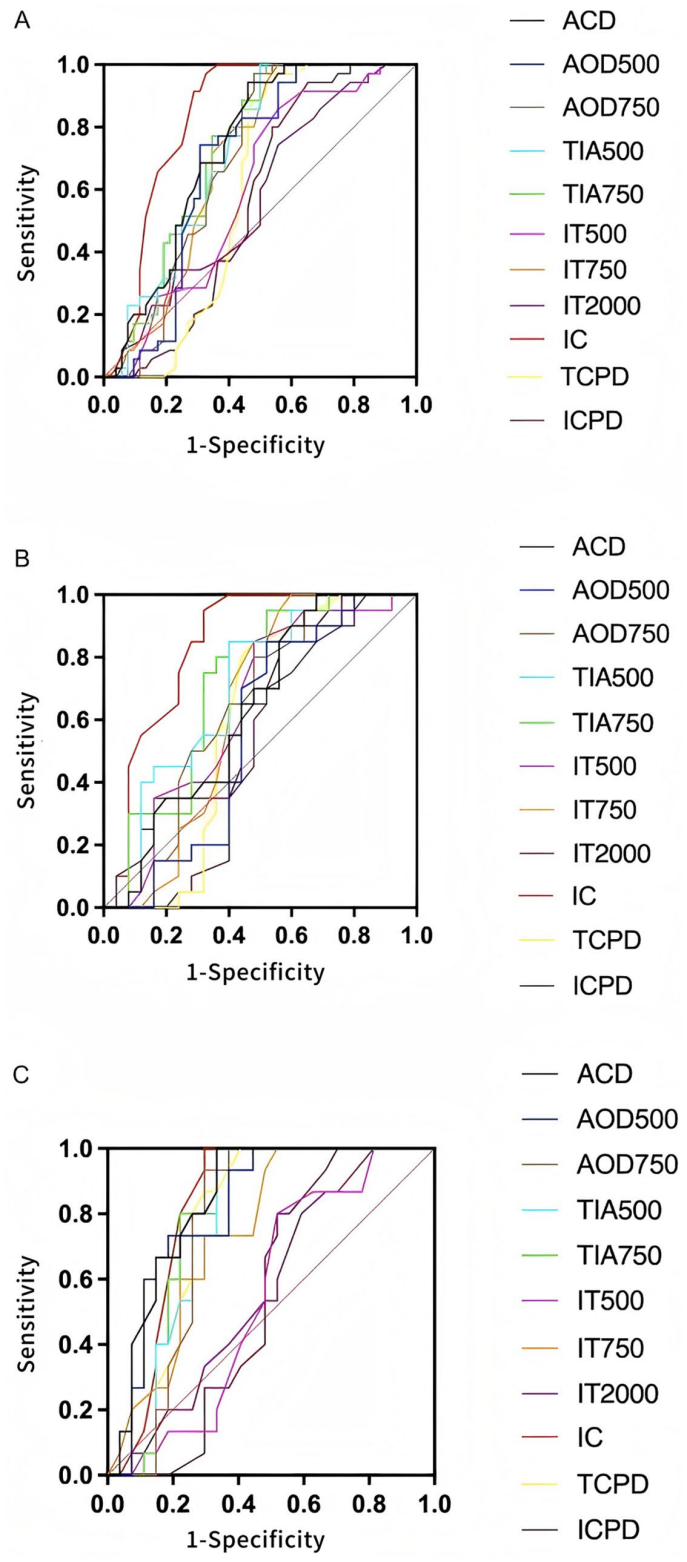
ROC curve analysis of various anterior segment parameters for identifying contralateral eyes of NVG. **(A)** Comparison of ROC curves for anterior segment parameters between the observation group and the control group. Parameters such as IC, ACD, and AOD750 demonstrated high diagnostic efficacy. **(B)** ROC curves between the young observation group and the young control group. The overall discriminatory ability of parameters was reduced, with only IC showing slight diagnostic value. **(C)** Comparison between the old observation group and the old control group. Most parameters exhibited good sensitivity and specificity, particularly ACD, IC, AOD500, and TIA750.

To assess the discriminative efficacy of anterior segment parameters in identifying early pathological changes in NVG fellow eyes, ROC curve analyses were performed, and stratified comparisons across age subgroups were conducted. Overall, most parameters demonstrated varying diagnostic capabilities between the observation and control groups. IC exhibited the best performance (AUC = 0.834, *p* < 0.05), indicating good diagnostic value for iris root anterior displacement in anatomical changes of NVG fellow eyes. Other parameters, including AOD750 (AUC = 0.690, *p* < 0.05), TIA750 (AUC = 0.726, *p* < 0.05), and ACD (AUC = 0.733, *p* < 0.05), showed moderate diagnostic efficacy, suggesting good sensitivity to structural changes in anterior chamber angle openness and depth during disease progression. In contrast, IT2000 (AUC = 0.574, *p* = 0.243) and ICPD (AUC = 0.561, *p* = 0.334) demonstrated weaker discriminative power, likely due to low sensitivity to pathological changes or significant inter-individual variation. In the younger subgroup, most parameters had lower AUC values compared to the overall analysis, with many failing to reach statistical significance. Notably, AOD750 (AUC = 0.642, *p* = 0.112) and TIA750 (AUC = 0.647, *p* = 0.063) were less effective, while only IC retained statistical significance (AUC = 0.684, *p* = 0.048). These findings suggest more subtle structural changes in younger populations, necessitating multi-parameter combinations or dynamic observation for improved diagnosis. In the older subgroup, several parameters showed good discriminative performance. ACD (AUC = 0.844, *p* < 0.05), AOD500 (AUC = 0.817, *p* < 0.05), TIA750 (AUC = 0.775, *p* < 0.05), and IC (AUC = 0.826, *p* < 0.05) achieved high statistical significance, indicating more pronounced angle structural changes and providing clearer diagnostic insights. IT750 (AUC = 0.747, *p* < 0.05) also showed good diagnostic performance, suggesting IT as a valuable parameter in older populations. However, distal iris parameters such as ICPD and IT2000 demonstrated limited clinical relevance across all groups, highlighting their restricted practical value in early-stage anatomical assessment. We performed ROC curve analysis for key anterior segment parameters. The AUC values, along with 95% confidence intervals (CIs), and Youden-based thresholds with corresponding sensitivity and specificity are summarized below: IC: AUC = 0.834 (95% CI: 0.766–0.902), Youden-based threshold = 0.210 mm, sensitivity = 84%, specificity = 75%. AOD750: AUC = 0.690 (95% CI: 0.608–0.772), Youden-based threshold = 0.320 mm, sensitivity = 72%, specificity = 63%. TIA750: AUC = 0.726 (95% CI: 0.636–0.815), Youden-based threshold = 26.5°, sensitivity = 75%, specificity = 68%. ACD: AUC = 0.733 (95% CI: 0.651–0.815), Youden-based threshold = 2.30 mm, sensitivity = 70%, specificity = 72%” (see Table 8 and Figure 2).

**Table 8:** Comparison of AUC values of anterior segment parameters between observation and control groups.

Parameter	Observation group vs. control group	Young observation group vs. young control group	Old observation group vs. old control group
ACD	AUC = 0.733 (95% CI: 0.651–0.815), *p* < 0.05	AUC = 0.630 (95% CI: 0.520–0.740), *p* = 0.138	AUC = 0.844 (95% CI: 0.752–0.936), *p* < 0.05
AOD500	AUC = 0.686 (95% CI: 0.589–0.783), *p* < 0.05	AUC = 0.652 (95% CI: 0.522–0.782), *p* = 0.479	AUC = 0.817 (95% CI: 0.731–0.903), *p* < 0.05
AOD750	AUC = 0.690 (95% CI: 0.594–0.786), *p* < 0.05	AUC = 0.642 (95% CI: 0.502–0.781), *p* = 0.112	AUC = 0.765 (95% CI: 0.664–0.865), *p* < 0.05
TIA500	AUC = 0.718 (95% CI: 0.627–0.809), *p* < 0.05	AUC = 0.643 (95% CI: 0.497–0.788), *p* = 0.073	AUC = 0.773 (95% CI: 0.675–0.871), *p* < 0.05
TIA750	AUC = 0.726 (95% CI: 0.633–0.819), *p* < 0.05	AUC = 0.647 (95% CI: 0.503–0.791), *p* = 0.063	AUC = 0.775 (95% CI: 0.676–0.874), *p* < 0.05
IT500	AUC = 0.603 (95% CI: 0.504–0.701), *p* = 0.105	AUC = 0.643 (95% CI: 0.487–0.799), *p* = 0.102	AUC = 0.551 (95% CI: 0.419–0.682), *p* = 0.590
IT750	AUC = 0.695 (95% CI: 0.603–0.787), *p* < 0.05	AUC = 0.634 (95% CI: 0.493–0.775), *p* = 0.126	AUC = 0.747 (95% CI: 0.646–0.849), *p* < 0.05
IT2000	AUC = 0.574 (95% CI: 0.468–0.679), *p* = 0.243	AUC = 0.588 (95% CI: 0.460–0.717), *p* = 0.315	AUC = 0.570 (95% CI: 0.421–0.718), *p* = 0.454
IC	AUC = 0.834 (95% CI: 0.766–0.902), *p* < 0.05	AUC = 0.684 (95% CI: 0.519–0.848), *p* = 0.048	AUC = 0.826 (95% CI: 0.748–0.904), *p* < 0.05
TCPD	AUC = 0.599 (95% CI: 0.492–0.706), *p* = 0.119	AUC = 0.596 (95% CI: 0.438–0.754), *p* = 0.273	AUC = 0.770 (95% CI: 0.651–0.889), *p* < 0.05
ICPD	AUC = 0.561 (95% CI: 0.453–0.669), *p* = 0.334	AUC = 0.537 (95% CI: 0.398–0.676), *p* = 0.673	AUC = 0.546 (95% CI: 0.398–0.693), *p* = 0.627

## 4 Discussion

The NVG is a severe blinding ocular disease secondary to retinal ischemic conditions, characterized by a complex pathogenesis, rapid progression, and poor prognosis. Although NVG typically manifests unilaterally at onset, clinical practice suggests that the contralateral eye often exhibits underlying high-risk structural alterations—particularly when the underlying pathology is bilateral. Thus, the potential for disease development in the contralateral eye cannot be overlooked. Structural monitoring of the contralateral eye in NVG patients holds significant clinical importance, especially before typical clinical symptoms emerge. Early detection of anatomical changes may enable timely intervention, potentially delaying or preventing disease progression. This study focused on alterations in the anterior segment structure of the contralateral eyes in NVG patients, with particular emphasis on quantitative parameters including ACD, AOD500/750, TIA500/750, IT500/750/2000, IC, and TCPD, to systematically evaluate the extent of structural abnormalities. Furthermore, age-stratified analysis was conducted to explore the potential modulating effect of age on anterior segment changes, providing data support for individualized risk assessment. Quantitative assessment of anterior segment anatomy—as a potential biomarker for NVG development and progression—may offer reliable imaging-based evidence for clinical use and facilitate the establishment of early screening and intervention strategies for high-risk populations. Therefore, this study has clear clinical relevance and practical value, providing a foundation for recognizing imaging characteristics and investigating pathological mechanisms in the contralateral eyes of NVG patients.

This study found that the contralateral eyes of NVG patients already exhibit significant anatomical differences in anterior segment structures compared to normal control eyes, suggesting that early structural alterations may be present even in the not-yet-affected contralateral eyes. The ACD in the observation group was significantly shallower than that in the control group. Angle opening parameters, including AOD500, AOD750, TIA500, and TIA750, were also significantly reduced, demonstrating morphological characteristics of a shallow anterior chamber and narrow angle. These anatomical changes may provide a structural basis for subsequent pathological processes such as impaired aqueous humor outflow and elevated IOP (12, 14, 15). The IC in the observation group was significantly higher than that in the control group, suggesting a possible anterior displacement of the iris root or compression due to changes in posterior chamber pressure from the ciliary body, further contributing to angle narrowing or even closure (16–19). ROC curve analysis results showed that the AUC values for IC, ACD, and AOD750 were all greater than 0.7, indicating strong discriminatory power. This suggests that these anterior segment parameters have high sensitivity and specificity in distinguishing between contralateral eyes of NVG patients and normal eyes, holding potential value for early screening. These findings further validate that quantitative assessment of anterior segment parameters using non-contact imaging techniques can enable the early identification of high-risk individuals, providing feasible structural biomarkers for subsequent intervention. Age subgroup analysis further revealed the age-dependent characteristics of anterior segment structural changes. In the older age subgroup (>60 years), the contralateral eyes of NVG patients exhibited more pronounced narrow anterior segment structures compared to age-matched normal controls, including a significantly shallower ACD, significantly decreased AOD500/750 and TIA500/750, and a significantly increased IC. This suggests that with increasing age, physiological degeneration of the anterior segment anatomy may exacerbate the expression of NVG-related pathological changes, making structural abnormalities more likely to manifest (15, 20, 21). ROC curve analysis also indicated that the aforementioned parameters had higher diagnostic efficacy in the older age group, with the AUC values of several indicators exceeding 0.75, particularly ACD, AOD750, and IC showing strong discriminatory ability. In the younger subgroup, although some parameters also changed, the intergroup differences were not significant, and the overall performance of the ROC curves was suboptimal, with only IC showing some value in discrimination. These results suggest that age not only influences the baseline state of anterior segment structures but may also act as an independent modulator of structural remodeling in the contralateral eyes of NVG patients, with anatomical-physiological degeneration and pathological progression potentially having synergistic effects. Therefore, age should be fully considered in anterior segment structural assessment and risk determination to develop more precise stratified diagnostic models. Correlation analysis among the parameters in this study revealed that the angle opening degree indicator AOD500 was significantly positively correlated with ACD, AOD750, and TIA500/750, indicating that a wider angle is typically accompanied by a deeper anterior chamber and expansion of the angle morphology, representing a comprehensive reflection of an unobstructed anterior segment state. Conversely, AOD500 was negatively correlated with IT750 and IC, suggesting that in a state of increased angle opening, the iris tends to be thinner and exhibit a posterior displacement trend, reflecting anatomical features of reduced iris tension or relatively stable posterior chamber pressure. This negative correlation was more pronounced in the older age subgroup, especially the negative correlation between AOD500 and IC, which was stronger. This indicates that the anterior segment structures in the elderly population are more sensitive to pathological load in terms of anatomical changes, conferring a higher risk of angle closure and suggesting a greater structural susceptibility to glaucoma in this population. This finding highlights the differences in anterior segment structural response mechanisms across different age groups, providing a theoretical basis for constructing age-specific anterior segment risk assessment systems in the future.

This study, as a single-center, retrospective, case–control cross-sectional investigation, although efforts were made to control for biases in study design and data analysis, its inherent limitations cannot be overlooked. Firstly, the relatively limited sample size, particularly within the age-stratified subgroups, raises the possibility of insufficient statistical power for differences in some parameters, affecting the extrapolation and generalizability of the results. Therefore, future studies are needed to further validate the conclusions based on multi-center, large-sample data. Secondly, the lack of longitudinal follow-up data in this study prevents the clarification of the temporal evolution of anterior segment structural changes and their causal relationship with the actual onset of NVG. Prospective cohort studies are required to further elucidate their predictive value. Furthermore, the anterior segment parameters used in this study highly depend on the acquisition quality of UBM images and the technical skill of the operator, introducing a certain risk of measurement errors and subjective judgment bias. Consequently, future research urgently needs to optimize the image acquisition process and establish standardized operating procedures and quality control systems to ensure data reproducibility and accuracy, thereby providing a more robust technical foundation for the quantitative assessment of anterior segment structures.

Future research should focus on the dynamic evolution of anterior segment structural changes to establish a systematic monitoring and risk assessment framework for the early identification and precise intervention of pathologies in the contralateral eyes of NVG patients. On the technical front, it is recommended to develop a multimodal dynamic monitoring model based on combined UBM and SS-OCT imaging, and to incorporate artificial intelligence-based image recognition and parameter extraction technologies to enhance the automation and sensitivity of anterior segment assessment, facilitating long-term longitudinal follow-up in clinical practice. Regarding the construction of an assessment system, integrating current morphological indicators such as angle width, iris configuration, and ACD with aqueous humor dynamics parameters could be attempted to create a comprehensive risk scoring model that combines structure and function, providing a higher-precision tool for predicting NVG risk. Simultaneously, the study population should be expanded to include high-risk groups such as patients with DR and those post-proliferative diabetic retinopathy (PDR) surgery, exploring the characteristics of structural evolution in their contralateral eyes under different pathological backgrounds. This will enable the development of differentiated monitoring and intervention strategies, thereby promoting the individualized and proactive development of early NVG prevention and control strategies.

## 5 Conclusion

Based on UBM, this study conducted a quantitative assessment of the anterior segment structures in the contralateral eyes of patients with unilateral NVG. The results revealed significant deviations from normal morphological characteristics in multiple parameters, including ACD, angle width, and iris concavity, suggesting that although these eyes are clinically “undiseased,” they may already be in a state of high anatomical risk. Age-stratified analysis further indicated that anatomical degeneration of the anterior segment is more pronounced in the elderly population, exhibiting a synergistic exacerbation trend with the structural changes in the contralateral eyes of NVG patients. ROC curve analysis demonstrated that parameters such as IC, ACD, and AOD750 possess high diagnostic efficacy in identifying high-risk contralateral eyes. Correlation analysis confirmed significant interrelated relationships between angle width indicators and other anterior segment structural parameters, suggesting that angle width may serve as a core parameter reflecting the comprehensive status of the anterior segment. In summary, quantitative changes in anterior segment structures may provide important imaging evidence for the early identification of contralateral eyes at risk of NVG. Combined multi-parameter analysis holds promise for constructing a prospective early-warning model to guide the development of individualized risk screening and dynamic management strategies.

## Data Availability

The original contributions presented in the study are included in the article/supplementary material, further inquiries can be directed to the corresponding author.
